# Age-Adjusted Cystatin C Z-Scores in Neonates and Infants

**DOI:** 10.1016/j.ekir.2025.09.008

**Published:** 2025-09-04

**Authors:** Guido Filler, Mashael Abujabal, Ajay Parkash Sharma, Joanne Grimmer, Amrit Kirpalani, Maria Esther Díaz González de Ferris

**Affiliations:** 1 Department of Paediatrics, Schulich School of Medicine and Dentistry, University of Western Ontario, London, Ontario, Canada; 2 Department of Medicine, Schulich School of Medicine and Dentistry, University of Western Ontario, London, Ontario, Canada; 3 Lilibeth Caberto Kidney Clinical Research Unit, London Health Sciences Centre, London, Ontario, Canada; 4 Children’s Health Research Institute, University of Western Ontario, London, Ontario, Canada; 5 Department of Pediatrics, East Jeddah General Hospital, Ministry of Health, Jeddah, Saudi Arabia; 6 Department of Pediatrics, University of North Carolina at Chapel Hill, Chapel Hill, North Carolina, USA

## Introduction

Diagnosing acute kidney injury (AKI) in neonates and infants remains a clinical challenge because of the limitations of current consensus definitions.^1^ The widely accepted Kidney Disease: Improving Global Outcomes criteria require an increase in serum creatinine by ≥ 0.3 mg/dl or oliguria, both of which are poorly suited to the physiology of early life.^2^ Creatinine is maternally transferred, insensitive to acute nephron loss, and influenced by body composition.^1^ Moreover, infants born with congenital renal hypoplasia may not show meaningful changes in serum creatinine even when function declines.

Cystatin C offers a physiologically grounded alternative. It is freely filtered by the glomerulus, not secreted, and unaffected by muscle mass or maternal levels.^3^ Although it is increasingly adopted in pediatric nephrology, it has not yet been incorporated into AKI definitions for neonates and infants because of the absence of a validated, age-adjusted reference framework.

To generate age-adjusted reference values, we applied the LMS method, a statistical technique widely used in growth-curve modeling. This approach characterizes age-related distributions using 3 parameters—lambda (L, skewness), mu (M, median), and sigma (S, coefficient of variation)—to construct smooth centile curves and corresponding z-scores across infancy. Full methodological details, including data harmonization, LMS modeling, and calculator implementation, are provided in the Supplementary Methods.

## Results

**Figure 1:**
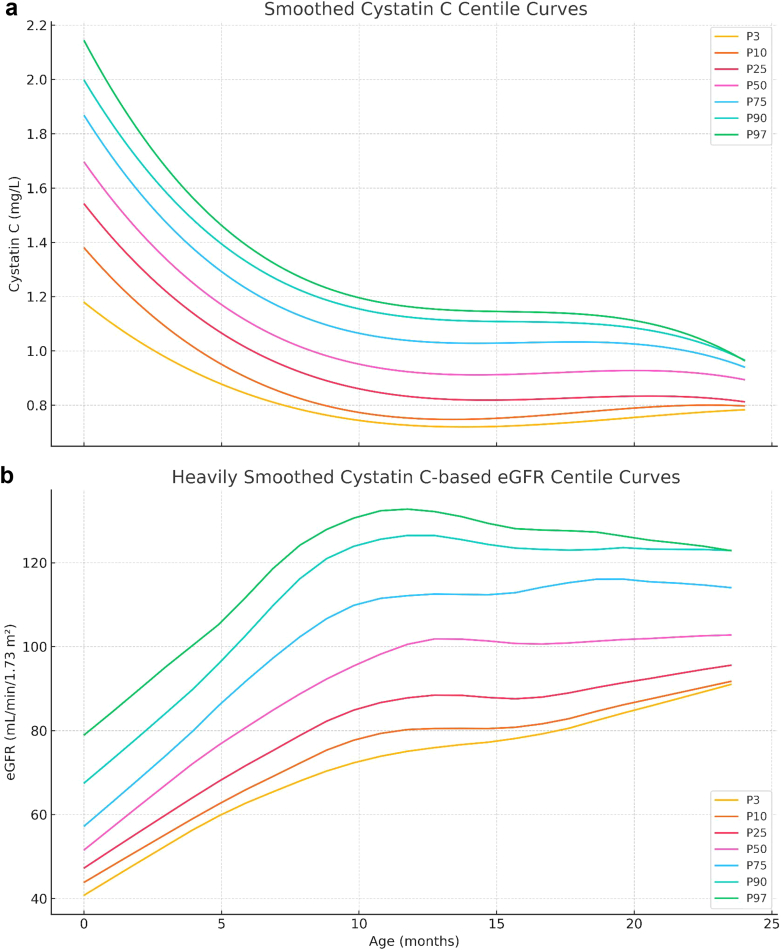
(a) Age-dependent percentile curves for serum cystatin C (mg/l) from 0 to 24 months. Curves represent the 3rd, 10th, 25th, 50th, 75th, 90th, and 97th percentiles derived from LMS modeling of 459 healthy infants. The progressive decline reflects the maturation of glomerular filtration. (b) Age-dependent percentile curves for cystatin C-based estimated glomerular filtration rate (eGFR, ml/min per 1.73 m^2^). Percentiles were derived from LMS modeling of eGFR values calculated via the Filler formula. The physiologic increase in eGFR is apparent across the first 2 years of life.

Median cystatin C declined from 1.86 mg/l at birth^4^ to 0.72 mg/l at 24 months, with corresponding estimated glomerular filtration rate (eGFR)^5^ increasing from 30.2 to 103.2 ml/min per 1.73 m^2^ (Supplementary Table S1 and Figure 1). The resulting LMS-based percentiles and z-scores accurately capture physiologic renal maturation across infancy (Supplementary Tables S2 and S3). We propose that a decline in eGFR z-score ≥ 0.5 represents possible AKI, whereas a drop ≥ 1.0 suggests likely AKI. These thresholds account for biological variability and assay imprecision (∼ ±8%) and are summarized in Table 1.

**Table 1:** Interpretation of cystatin C z-score decline

Δ z-score	Interpretation	Suggested action
< −0.5	Stable function	Routine monitoring
−0.5 to −0.9	Possible early AKI	Clinical correlation advised
≥ −1.0	Likely AKI	Initiate workup and intervention

AKI, acute kidney injury.

## Discussion

Our approach addresses a long-standing diagnostic blind spot in neonatology and pediatric nephrology. By relying on developmental physiology rather than fixed serum creatinine thresholds, cystatin C–eGFR z-scores allow clinicians to identify renal functional decline even when absolute values appear “normal.”

Our normative datasets originated from 2 Canadian centers (London and Ottawa) and were harmonized to ERM-DA471/IFCC standards, minimizing assay-related bias. Both cohorts were highly comparable in design and recruitment. Nonetheless, we acknowledge that regional and ethnic differences in kidney function cannot be excluded, and validation of this z-score framework in broader populations will be important to confirm global applicability. In addition, the clinical use of cystatin C requires careful attention to assay methodology. Standardization to ERM-DA471/IFCC reference material is essential, and results from nontraceable platforms may require correction factors before integration into z-score calculations. This aligns with current IFCC recommendations and ensures comparability across laboratories.

This framework enables the detection of AKI superimposed on baseline chronic kidney disease. A decline in cystatin C–eGFR z-score > 0.5 may indicate early AKI, whereas a decline > 1.0 suggests likely AKI. It may facilitate earlier recognition of congenital anomalies of the kidney and urinary tract or hereditary nephropathies, such as autosomal polycystic kidney disease. In addition, the tool enables forward prediction of expected renal function at 18 to 24 months, supporting early prognosis and intervention planning.

This method may inform the evolving definition of pediatric chronic kidney disease. Current Kidney Disease: Improving Global Outcomes guidelines require ≥3 months of reduced function to diagnose chronic kidney disease, yet this timeline is impractical for newborns with congenital kidney disease. A z-score-based system may enable earlier recognition of nontransient disease based on impaired maturation trajectories.

Importantly, this approach aligns with NIH/FDA ADEPT 8 priorities for developing lifespan-aware frameworks that transcend binary pediatric/adult distinctions.^6^ The tool may support early AKI biomarker validation, pharmacokinetic modeling, and individualized nephrotoxic exposure monitoring in neonatal intensive care unit settings.

In addition, recent work has shown that discordance between creatinine- and cystatin C–based eGFR may reflect differences in nutritional status and body composition.^7^ This is particularly relevant in infants with growth faltering, where cystatin C–eGFR z-scores may provide a more physiologically accurate assessment of kidney function than creatinine.

Cystatin C–eGFR z-scores provide a developmentally appropriate, laboratory-based method to detect AKI and predict chronic kidney disease in neonates and infants. This approach offers a pragmatic advance in early-life nephrology and may reshape diagnostic frameworks in this vulnerable and often overlooked population.

## Disclosure

All the authors declared no competing interests.

## Supplementary Material

Supplementary File (PDF) **Supplementary Methods. Supplementary References. Table S1.** Basic statistics of the participant cohort. **Table S2.** LMS parameters for cystatin C. **Table S3.** LMS parameters for cystatin C eGFR. STROBE checklist.
