# Supplementary material for: Age-Adjusted Cystatin C Z-Scores in Neonates and Infants
Source: Kidney Int Rep. 2025 Sep 4;10(11):4087–9. doi: 10.1016/j.ekir.2025.09.008 (PMC12640028; doi:10.1016/j.ekir.2025.09.008)
Supplement: Supplementary File (PDF) — Supplementary Methods. Supplementary References. Table S1. Basic statistics of the participant cohort. Table S2. LMS parameters for cystatin C. Table S3. LMS parameters for cystatin C eGFR. STROBE checklist. [file mmc1.pdf]

## Supplementary Methods

### *Patient population*

This study utilized two previously published datasets comprising serum cystatin C concentrations in healthy neonates and infants. The neonatal cohort (*Bariciak et al.*) included 128 term newborns recruited at The Ottawa Hospital in Canada.[4] These infants were assessed on postnatal days 1 and 3, had no perinatal complications, and were considered clinically well by their neonatologists. All samples were collected with parental consent and approved under the original research ethics protocol.

The second dataset (*Filler et al.*) was drawn from a larger cohort of 331 healthy infants aged between 0.2 and 24 months, recruited during clinic visits at London, Ontario, Canada, for minor issues such as minimal dilatation of the renal pelvises.[3] Most newborns had two measurements between days one and three of life, resulting in a total of 551 measurements across 459 participants. Exclusion criteria included prematurity (gestational age <37 weeks), known congenital anomalies of the kidney and urinary tract, and any acute illness at the time of testing. Only children with normal weight and length percentiles and no reported renal, cardiac, or systemic disorders were included. No adjustments were made for sex, as previous studies have shown minimal sex-based variation in cystatin C production during infancy. Ethnicity data were not consistently available and were therefore excluded from the modeling.

### *Study setting*

The neonatal cohort was recruited from The Ottawa Hospital, a tertiary perinatal center in Ontario, Canada. The infant cohort was recruited from outpatient clinics affiliated with the Children's Hospital at London Health Sciences Centre, a tertiary care center serving a catchment population of approximately four million across Southwestern Ontario. All laboratory analyses were conducted at affiliated hospital laboratories using validated clinical platforms.

For the current analysis, both datasets were combined into a single normative cohort after appropriate harmonization and adjustment of neonatal cystatin C values to align with the standardized reference material used in the later study. All cystatin C concentrations were measured using immunoassays traceable to ERM-DA471/IFCC, and eGFR was calculated using the Filler formula (eGFR = 91.62 / [cystatin C]^1.123).[S1] This study represents a secondary analysis of previously published, de-identified data and did not require additional research ethics board approval.

### *Missing data*

No imputation methods were applied. Nineteen neonates did not have blood samples collected on day 1 and day 3 of life. Only complete observations with valid cystatin C concentrations and age data were included in the LMS modeling. The neonatal dataset had minimal missing data due to

its prospective design. The infant dataset excluded cases with incomplete demographic or biochemical information.

### *Data modeling and z-score derivation*

The merged dataset of 459 infants was stratified by postnatal age. Age-specific reference values for serum cystatin C and eGFR were smoothed using the LMS method, which models three age-dependent parameters: L (skewness), M (median), and S (coefficient of variation). LMS parameter estimation was performed using a Box-Cox, Cole, and Green approach with penalized likelihood and cubic spline fitting to minimize overfitting while ensuring physiological plausibility.

Separate LMS models were generated for cystatin C and eGFR values using R (version 4.3) and the `gamlss` package. Percentile curves corresponding to the 3rd, 10th, 25th, 50th, 75th, 90th, and 97th percentiles were plotted. For clinical usability, an Excel-based z-score calculator was developed, enabling clinicians to enter the date of birth, test date, and cystatin C concentration, and receive eGFR values and corresponding z-scores. The z-score was calculated using the standard LMS equation:

$$Z = \frac{(Y/M(t))^{L}(t) - 1}{L(t).S(t)} if\ L \neq 0\ else\ Z = \frac{\ln{(Y/M(t))}}{S(t)}$$

### *Excel calculator implementation*

To ensure accessibility in routine clinical practice, an interactive Excel spreadsheet was created. The calculator requires three user inputs: (1) date of birth, (2) date of cystatin C measurement, and (3) cystatin C concentration in mg/L. Age in months is calculated automatically using Excel's `DATEDIF` function. The spreadsheet then uses a `VLOOKUP` function to retrieve the corresponding L, M, and S parameters from the LMS table based on age. eGFR is calculated using the Filler formula, and the z-score is derived using the LMS equation embedded in the worksheet. Output cells display the eGFR (mL/min/1.73 $m^2$), z-score, and the corresponding percentile classification, with conditional formatting to highlight potential AKI (e.g., z-score drop >1.0). The calculator supports serial assessments and is adaptable for electronic health record integration.

The LMS values for cystatin C and cystatin C-based eGFR were calculated across age bins of varying widths: 0.5-month intervals from birth to 3.5 months, 1-month intervals between 4.5 and 6.5 months, and 2-month Intervals from 8 to 22 months.

### *Assessment and mitigation of measurement bias*

To ensure comparability between the neonatal and infant datasets, a bias harmonization step was undertaken based on known assay differences. The Bariciak cohort utilized the Dade Behring cystatin C assay via nephelometry, which is known to yield values 10% higher than those obtained with standardized immunoassays aligned with the ERM-DA471/IFCC reference material.[4] This

bias was addressed by applying a 10% downward correction. Consistent with published calibration data and the observed inter-method discrepancy at the time of transition to standardized assays. The Filler et al. dataset utilized the Roche immunoassay, which was calibrated to certified reference materials as described by Ismail and Filler (2017)[S2] and therefore required no adjustment. The neonatal dataset had the advantage of uniform sample timing (day 1 and 3) and prospective recruitment in a tightly controlled hospital setting, potentially reducing interindividual variability and residual confounding.

### *Data sharing*

The de-identified dataset analyzed in this study is available from the corresponding author upon request. The Excel-based calculator for cystatin C-derived eGFR z-score computation is also available upon request for academic and non-commercial use. All requests will be reviewed in accordance with institutional data-sharing policies.

### *Supplementary References*


S1. Filler G, Lepage N. Should the Schwartz formula for estimation of GFR be replaced by cystatin C formula? *Pediatr Nephrol* 2003; **18:** 981-985.

S2. Ismail OZ, Bhayana V, Kadour M*, et al.* Improving the translation of novel biomarkers to clinical practice: The story of cystatin C implementation in Canada: A professional practice column. *Clin Biochem* 2017; **50:** 380-384.

**Supplementary Table S1. Basic statistics of the participant cohort.**

| Variable | Mean | Median | Standard Deviation | IQR (Q1, Q3) | Minimum | Maximum |
|---|---|---|---|---|---|---|
| Age months | 6.16 | 2.00 | 7.69 | (0.1, 11.0) | 0.02 | 24.00 |
| Cystatin_C | 1.35 | 1.34 | 0.41 | (1.02, 1.66) | 0.65 | 2.93 |
| eGFR | 72.92 | 66.0 | 25.8 | (51.9, 89.6) | 27.4 | 148.6 |

**Supplementary Table S2. LMS parameters for cystatin C.**

| Age (months) | L | M | S |
|---|---|---|---|
| 0 | 0.376009 | 1.68 | 0.175 |
| 0.5 | 2.424161 | 1.55 | 0.141067 |
| 1.5 | 1.886832 | 1.51 | 0.151118 |
| 2.5 | 0.194542 | 1.3 | 0.122604 |
| 3.5 | 1.445342 | 1.235 | 0.130836 |
| 4.5 | 1.330652 | 1.16 | 0.171401 |
| 5.5 | -0.08593 | 1.015 | 0.191273 |
| 6.5 | 1.466947 | 1.1 | 0.146253 |
| 8 | 0.44841 | 0.96 | 0.18692 |
| 10 | 0.889437 | 0.93 | 0.158282 |
| 12 | 0.227132 | 0.92 | 0.15256 |
| 14 | -0.53211 | 0.9 | 0.186127 |
| 16 | 0.030315 | 0.89 | 0.143169 |
| 18 | 2.007818 | 0.955 | 0.145439 |
| 20 | 0.777839 | 0.88 | 0.135008 |
| 22 | 3.06401 | 0.96 | 0.077888 |

**Supplementary Table S3. LMS parameters for cystatin C eGFR.**

| Age (months) | L | M | S |
|---|---|---|---|
| 0 | -0.33482 | 51.16441 | 0.210785 |
| 0.5 | -2.15865 | 56.01174 | 0.204237 |
| 1.5 | -1.68017 | 57.67783 | 0.204106 |
| 2.5 | -0.17323 | 68.24522 | 0.13797 |
| 3.5 | -1.28704 | 72.29935 | 0.160833 |
| 4.5 | -1.18491 | 77.55569 | 0.220385 |
| 5.5 | 0.076522 | 90.12634 | 0.201085 |
| 6.5 | -1.30627 | 82.32202 | 0.192165 |
| 8 | -0.3993 | 95.92005 | 0.220981 |
| 10 | -0.79202 | 99.40166 | 0.203617 |
| 12 | -0.20225 | 100.6158 | 0.169354 |
| 14 | 0.473833 | 103.1909 | 0.194024 |
| 16 | -0.02699 | 104.4323 | 0.148395 |
| 18 | -1.78791 | 96.48736 | 0.199341 |
| 20 | -0.69264 | 105.9127 | 0.157477 |
| 22 | -2.72842 | 95.92005 | 0.097528 |

STROBE Statement Checklist

Title and abstract:

1(a) Study design indicated: Observational cohort study clearly indicated, but abstract had to be deleted for research letter.

1(b) Balanced summary provided: Yes, informative and balanced summary in abstract.

Introduction:

2. Background and rationale clearly explained: Clearly explained in the introduction.
3. Objectives and prespecified hypotheses clearly stated: Clearly stated in the introduction.

Methods:

4. Study design clearly described: Clearly described in the methods section as secondary analysis of prospectively collected data.
5. Setting, location, dates clearly described: Clearly described—Ottawa and London, Canada.

6(a). Eligibility criteria and sources/methods of participant selection clearly described: Clearly described in methods (healthy term neonates and infants).

6(b). Matching criteria: Not applicable (N/A).

7. Outcomes, exposures, predictors, confounders clearly defined: Clearly defined (Cystatin C and eGFR measures, demographic criteria).
8. Sources of data and assessment methods clearly described: Clearly described (Cystatin C measured via standardized assays).
9. Efforts to address potential sources of bias clearly described: Clearly described (harmonization across assays).
10. Study size clearly explained: Clearly described (459 infants, 551 measurements).
11. Handling of quantitative variables clearly described: Clearly described (LMS method, z-scores).

12(a). Statistical methods clearly described: Clearly described (LMS, cubic spline).

12(b). Subgroup analyses clearly described: Not applicable.

12(c). Missing data clearly described: Clearly described, no imputation used.

12(d). Follow-up clearly described: Not applicable.

12(e). Sensitivity analyses clearly described: Not conducted, clearly noted.

Results:

13(a). Numbers of individuals at each stage clearly reported: Clearly reported (459 infants, 551 measurements).

13(b). Reasons for non-participation clearly reported: Minimal missing data described.

13(c). Flow diagram provided: Not included, not necessary due to secondary analysis.

14(a). Participant characteristics clearly provided: Clearly provided (demographics, age, cystatin C levels, eGFR values).

14(b). Missing data per variable clearly reported: Clearly reported (minimal missing data).

14(c). Follow-up time summarized: Not applicable.

15. Outcome data clearly provided: Clearly provided (median and IQR reported for eGFR and cystatin C).

16(a). Main results clearly reported with estimates: Clearly reported (centile curves, median eGFR and cystatin C values).

16(b). Category boundaries clearly reported: Clearly reported (age bins described).

16(c). Absolute risks clearly reported: Not applicable.

17. Other analyses clearly reported: Not conducted.

Discussion:

18. Key results clearly summarized: Clearly summarized relative to study objectives.
19. Limitations clearly discussed: Clearly discussed (missing race/ethnicity data, assay variability).
20. Overall interpretation cautious and balanced: Clearly cautious, balanced, and well-reasoned.
21. Generalizability clearly discussed: Clearly discussed, with suggestions for broader validation.

Other Information:

22. Funding sources clearly described: Clearly stated, no external funding.
